# Respiratory Syncytial Virus Vaccination in Allogeneic Hematopoietic Stem Cell Transplant Recipients

**DOI:** 10.1001/jamanetworkopen.2025.33828

**Published:** 2025-09-26

**Authors:** Rabah Redjoul, Christine Robin, Laurent Softic, Clément Ourghanlian, Ludovic Cabanne, Florence Beckerich, Amandine Caillault, Alexandre Soulier, Pauline Daumerie, Slim Fourati, Sébastien Maury

**Affiliations:** 1 Hematology Department, Henri Mondor Hospital, Assistance Publique-Hôpitaux de Paris, RedGene Federation, Créteil, France; 2 INSERM U955, Paris Est Créteil University UPEC, Créteil, France; 3 Virology Department, Henri Mondor Hospital, Assistance Publique-Hôpitaux de Paris, Créteil, France; 4 Pharmacy Department, Henri Mondor Hospital, Assistance Publique-Hôpitaux de Paris, Créteil, France

## Abstract

This cohort study evaluates immune response to respiratory syncytial virus (RSV) vaccination in allogeneic hematopoietic stem cell transplant recipients.

## Introduction

After allogeneic hematopoietic stem cell transplantation (HSCT), respiratory syncytial virus (RSV) infection can lead to severe infection with a poor prognosis.^1^ Three vaccines targeting the RSV prefusion F (pre-F) protein have been approved in US and Europe for adults aged 60 years or older, based on large randomized trials.^2,3,4^ As immunocompromised patients were excluded from these registration clinical trials, we aimed to evaluate vaccine immunogenicity in HSCT adult patients as a first estimation of its protective capacity in adults with such immune conditions.

## Methods

Between October 1 and November 21, 2024, 92 patients transplanted for myeloid (64 patients) or lymphoid (20 patients) malignant neoplasms (all but 3 in remission at HSCT) or nonmalignant disease (8 patients), who all shared risk factors for severe RSV infection (eMethods in Supplement 1), received bivalent pre-F RSV vaccine (Abrysvo [Pfizer]). We analyzed vaccine immunogenicity by quantifying serum anti–pre-F RSV IgG (eMethods in Supplement 1) at time of vaccination (baseline) and at 4 weeks after vaccination, as well as at 12 weeks for 47 patients.^2,4^ Major end points were the frequency and factors associated with seroconversion defined as 4-fold increase in antibody titers from baseline to 4 weeks after vaccination.^5^ This study was approved by the Paris Hospital Consortium Committee and by national Sud-Est VI ethics committee. Participants provided written informed consent. This study followed the guidelines of the Declaration of Helsinki and the Strengthening the Reporting of Observational Studies in Epidemiology (STROBE) reporting guideline. Categorical variables were compared by Fisher exact tests. Comparisons of continuous variable medians were performed using a Mann-Whitney test. Two-sided P values less than .05 were considered significant. Analyses were completed in GraphPad Prism 10.4.1 (GraphPad).

## Results

Among 92 participants, 61 (66%) were male, and the median (IQR) age at vaccination was 63 (53-70) years. Anti–pre-F IgG were detected and could be quantified at baseline in 82 recipients (89%) (median [IQR], 7582 [2170-19 288] IU/ml) while 10 (11%) were negative (<10 IU/ml). Lower titers at baseline correlated significantly with shorter interval after HSCT (Figure, A), low lymphocyte counts (Figure, B), and use of immunosuppressants within the 3 months preceding vaccination (Figure, C). No serious adverse events were observed after 1 or 2 vaccine doses. Longer time since HSCT, absence of systemic immunosuppressive drugs, and high lymphocyte counts significantly correlated with seroconversion (Table). Seroconversion occurred in 75% of patients (45 of 60) beyond 1 year following HSCT; this applied to only 9% of patients vaccinated within the first year (3 of 32). Intravenous immunoglobulin supplementation within 3 months preceding vaccination was more common in nonresponding patients, reflecting a higher level of immunosuppression. Notably, these same factors correlated with seroconversion when defined by a 2-fold lower or 8-fold higher number of anti-RSV titers. Six out of the 10 recipients showing undetectable anti–pre-F IgG at baseline had detectable anti-RSV titers at 4 weeks after vaccination.

**Figure.**
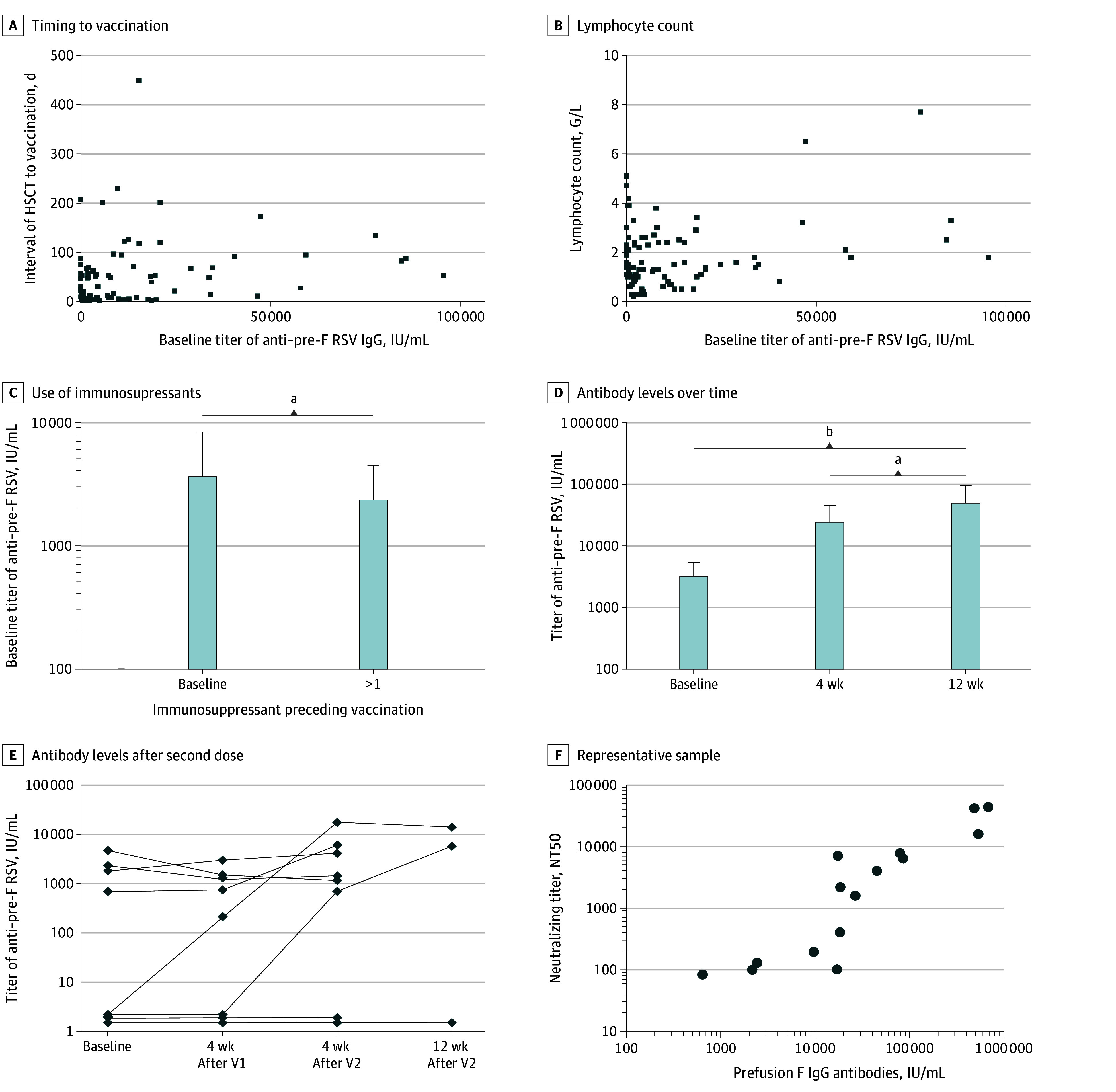
Antibody Response Among Allogeneic Hematopoietic Stem Cell Transplant Recipients Following Respiratory Syncytial Virus (RSV) Vaccination A, Spearman correlation between anti–pre-F RSV IgG baseline titers and timing between HSCT and vaccination (*r* = 0.211; *P* = .04). B, Spearman correlation between anti–pre-F RSV IgG baseline titers and lymphocyte count in peripheral blood at vaccination (*r* = 0.269; *P* = .01). C, Patients receiving at least 1 systemic immunosuppressant over the 3 months preceding vaccination (38 patients) show lower baseline anti–pre-F RSV IgG titers as compared with others (54 patients; *P* = .02). D, Anti-RSV IgG titers at 4 weeks (92 patients) and 12 weeks (47 patients) after 1 vaccine dose. Each box plot indicates the geometric mean value. Error bars indicate 95% CIs. Indicated statistical comparison are using a 1-way analysis of variance test for multiple comparisons. E, Humoral response in 8 patients who were not considered protected despite vaccination (see criteria in eMethods in Supplement 1) and therefore received a second vaccine dose in the 5 to 7 weeks following the first vaccine dose. F, Correlation analysis between normalized pre-F IgG antibodies and neutralizing antibody titers in 15 serum samples from representative samples of the cohort (*r* = 0.916; *P* < .0001). The samples have been selected to provide a representative range of different antibody levels. HSCT indicates hematopoietic stem cell transplantation; pre-F, prefusion-F; V1, first vaccination; V2, second vaccination. ^a^*P* < .05. ^b^*P* < .001.

**Table.** Characteristics of Patients Receiving Respiratory Syncytial Virus (RSV) Vaccination According to Antibody Response

Characteristic	Patients, No (%)			*P* value^b^
	All (N = 92)	Seroconversion^a^		
		No (n = 44)	Yes (n = 48)	
Demographics				
Sex				
Male	61 (66)	29 (66)	32 (67)	>.99
Female	31 (34)	15 (34)	16 (33)	
Age at transplant, median (IQR), y	60 (52-64)	57 (44-61)	61 (57-65)	.01
Age at vaccination, median (IQR), y	63 (53-70)	60 (45-65)	67 (62-73)	<.001
Transplant characteristic				
Donor type				
HLA-identical sibling	31 (34)	15 (34)	16 (33)	.27
Haplo-identical related	25 (27)	15 (34)	10 (21)	
Unrelated	36 (39)	14 (32)	22 (46)	
Posttransplant cyclophosphamide				
No	63 (68)	27 (61)	36 (75)	.18
Yes	29 (32)	17 (39)	12 (25)	
Acute GVHD grading^c^				
0	72 (78)	33 (75)	39 (81)	.80
I	4 (4)	2 (5)	2 (4)	
II	13 (14)	8 (18)	5 (10)	
III	2 (2)	1 (2)	1 (2)	
IV	1 (1)	0	1 (2)	
Chronic GVHD grading^d^				
No	68 (74)	33 (75)	35 (73)	.84
Mild	4 (4)	1 (2)	3 (6)	
Moderate	11 (12)	5 (11)	6 (12.5)	
Severe	9 (10)	5 (11)	4 (8)	
Interval between transplant and vaccination				
3-6 mo	18 (20)	16 (36)	2 (4)	<.001
7-12 mo	14 (15)	13 (30)	1 (2)	
>12 mo	60 (65)	15 (34)	45 (94)	
Immunosuppressants taken in the 3 mo preceding vaccination (%)				
Ciclosporin/sirolimus				
No	60 (65)	17 (39)	43 (90)	<.001
Yes	32 (35)	27 (61)	5 (10)	
Systemic steroids				
No	90 (98)	42 (95)	48 (100)	.23
Yes	2 (2)	2 (5)	0	
Ruxolitinib				
No	84 (91)	38 (86)	46 (96)	.15
Yes	8 (9)	6 (14)	2 (4)	
No. of immunosuppressants taken				
0	54 (59)	13 (30)	41 (85)	<.001
1	29 (32)	24 (54)	5 (10)	
2	8 (9)	6 (14)	2 (4)	
3	1 (1)	1 (2)	0	
Immune status at vaccination				
<3-mo IVIg supplementation (%)				
No	76 (83)	32 (73)	44 (92)	.02
Yes	16 (17)	12 (27)	4 (8)	
Lymphocytes in PB, median (IQR), Giga/L	1.5 (1.0-2.4)	1.1 (0.6-1.8)	2.1 (1.4-2.5)	.002
<1.4	40 (43)	29 (66)	11 (23)	<.001
≥1.4	52 (57)	15 (34)	37 (77)	

Abbreviations: GVHD, graft-vs-host disease; HLA, human leukocyte antigens; IVIg, intravenous immunoglobulin; PB, peripheral blood.

^a^ Defined as 4-fold or greater rise in IgG titer from baseline to 4 weeks (median [IQR], 30 [28-33] days) postvaccination. In the 10 patients showing undetectable anti–pre-F IgG at baseline (<10 IU/ml), the detection of anti–pre-F IgG at 4 weeks was considered a seroconversion (6 of 10 patients).

^b^ Categorical variables were compared by Fisher exact tests. Comparisons of continuous variables medians were performed using a Mann-Whitney test.

^c^ Grading according to Glucksberg scale.

^d^ Grading according to the National Institutes of Health classification global scoring.

In responding patients, high antibody levels remained over time, significantly higher at 12 weeks as compared with 4 weeks after vaccination (Figure, D). A second vaccine dose was administered in patients considered nonprotected despite vaccination (eMethods in Supplement 1). Among 8 revaccinated patients, 3 showed increased anti-RSV titers following the second vaccine dose that persisted elevated in the 2 recipients who were tested beyond 4 weeks following the second vaccine dose (Figure, E).

We further analyzed the correlation between RSV PreF-IgG antibodies titers with neutralizing antibody titers in a subset of patients. In 15 recipients selected to provide a representative range of different antibody levels, we saw a strong correlation between neutralizing antibody titers (50% neutralizing titer) and normalized RSV PreF-IgG antibodies (Figure, F).

## Discussion

In this cohort of immunocompromised individuals, patients vaccinated with 1 dose within the first year following HSCT had low rates of seroconversion. This parallels the very recent study^5^ of 38 immunocompromised participants of various conditions, where approximately 40% did not seroconvert or achieve a conservative neutralization threshold postvaccination. In overt nonresponding patients, we found that a second vaccine dose was associated with humoral immune response. Several clinical and biological factors previously associated with vaccine immunogenicity against other viruses in HSCT recipients^6^ were shared with regard to RSV. Limitations of the study include the absence of immunization quantification at long term after vaccination. Additional studies are also needed to define a clinical protection correlate in these unfavorable immune conditions and to conduct multivariable analyses, also taking into account the putative impact of cellular responses to vaccination.

## Notes

Supplement 1.
eMethods.

Supplement 2.
Data Sharing Statement
