# Supplementary material for: Respiratory Syncytial Virus Vaccination in Allogeneic Hematopoietic Stem Cell Transplant Recipients
Source: JAMA Netw Open. 2025 Sep 26;8(9):e2533828. doi: 10.1001/jamanetworkopen.2025.33828 (PMC12475948; doi:10.1001/jamanetworkopen.2025.33828)
Supplement: Supplement 1. — eMethods. [file jamanetwopen-e2533828-s001.pdf]

Supplemental Online Content

Redjoul R, Robin C, Softic L, et al. Respiratory syncytial virus vaccination in allogeneic hematopoietic stem cell transplant recipients. *JAMA Netw Open*. 2025;8(9):e2533828.
doi:10.1001/jamanetworkopen.2025.33828

eMethods.

This supplemental material has been provided by the authors to give readers additional information about their work.

eMethods

Clinical criteria to enter the vaccination program

Allogeneic hematopoietic stem cell transplantation (HSCT) patients showing at least one of the following criteria for high risk of severe RSV infection () were offered to be vaccinated as early as they had reached more than 3 months after HSCT:

- Age ≥60 years
- First year following HSCT
- Acute or chronic graft-versus-host disease (GVHD) treated with systemic immunosuppressive therapy
- Systemic steroids for non-GVHD reason
- Past or present history of pulmonary GVHD
- Chronic pulmonary and/or heart disease

Patients who received Rituximab in the 6 preceding months were not vaccinated due to the known negative impact on vaccinal immune responses in this setting. This was the sole exclusion criteria. Vaccination could be delayed as early as patients had reached more than 6 months after rituximab. Among the whole series of 92 patients, rituximab was given more than 6 months before vaccination in 6 patients (2 who seroconverted and 4 who did not).

Eligibility criteria for a second vaccine dose, corresponding to patients considered as nonprotected despite first vaccine dose:

- Undetectable anti-Pre-F IgG at baseline AND titer at 4 weeks post-vaccination below 1000 UI/ml,

OR

- Detectable anti-Pre-F IgG below 5000 UI/ml at baseline AND titer at 4 weeks post-vaccination below 5000 UI/ml and 2-fold basal level.

Serum anti-pre-F RSV IgG quantification

The immunological assay is based on the detection of IgG antibodies to RSV preF antigens in serum using a commercially available "Human Anti-RSV-F0 Antibody IgG titer ELISA Assay Kit" (ACRObiosystems) according to the manufacturer's recommendations. The assay is based on a standard indirect ELISA format. For validation and quantitation in IU/ml, serial dilutions of serum, negative and positive controls, and antiserum to Respiratory Syncytial Virus (WHO 1st International Standard, NIBSC 16/284) are used.

Neutralization assays

© 2025 Redjoul R et al. *JAMA Network Open*.

Neutralization assays used RSV-A (starin VR26 ATCC) infecting HEp-2 cells in the presence of serial dilutions of serum (1:4 to 1:2048). Twenty-four hours after infection, cells are immunostained using a mouse anti-RSV M2-1 monoclonal antibody colored in red.

Adverse events collection after receipt of one Single Dose of Abrysvo™, Pfizer Inc.

Solicited and unsolicited adverse events were self-reported through structured questionnaires given at vaccination and returned one month later. Of note, we did not observe any GVHD reactivation/flare-up following RSV vaccination.

© 2025 Redjoul R et al. *JAMA Network Open*.
