# Supplementary material for: Respiratory Syncytial Virus Vaccination in Allogeneic Hematopoietic Stem Cell Transplant Recipients
Source: JAMA Netw Open. 2025 Sep 26;8(9):e2533828. doi: 10.1001/jamanetworkopen.2025.33828 (PMC12475948; doi:10.1001/jamanetworkopen.2025.33828)
Supplement: Supplement 2. — Data Sharing Statement [file jamanetwopen-e2533828-s002.pdf]

# Data Sharing Statement

Redjoul. Respiratory Syncytial Virus Vaccination in Allogeneic Hematopoietic Stem Cell Transplant Recipients. *JAMA Netw Open*. Published September 26, 2025.
doi:10.1001/jamanetworkopen.2025.33828

**Data**
**Data available:** Yes
**Data types:** Deidentified participant data, Data dictionary
**How to access data:** on request to the corresponding author
**When available:** With publication

**Supporting Documents**
**Document types:** Statistical/analytic code, Informed consent form
**How to access documents:** on request to the corresponding author
**When available:** With publication

**Additional Information**
**Who can access the data:** on request to the corresponding author
**Types of analyses:** for a specified purpose
**Mechanisms of data availability:** after approval of a proposal and with a signed data access agreement
